# What Happens Next? Conducting Research With Cancer Survivors Who Discontinue Their Post-Treatment Care

**DOI:** 10.1093/geroni/igab046.090

**Published:** 2021-12-17

**Authors:** Aaron Seaman, Nitin Pagedar

**Affiliations:** University of Iowa, Iowa City, Iowa, United States

## Abstract

Due to improvements in screening, diagnosis, and treatment, more cancer patients are surviving and living longer. For them, survivorship care provides critical support: surveillance and screening for recurrence and new cancers; physical and psychological symptom management; social and financial management support; management of other chronic conditions; and preventive health and health promotion support. Yet, our pilot data indicates that a substantial number of survivors discontinue their survivorship care with the treating oncology team, a team that often provides critical multidisciplinary support and expertise. While it is important to understand the experiences, outcomes, and needs of these survivors, they can be challenging to engage in research. In this presentation, we will discuss survivors who discontinue, why they might do so, and methods for engaging them in research, drawing upon our work with head and neck cancer survivors.

